# Environmental prescribing for asthma and chronic obstructive pulmonary disease

**DOI:** 10.4102/phcfm.v18i1.5510

**Published:** 2026-08-13

**Authors:** Robert Mash, Helen Twohig

**Affiliations:** 1 Division of Family Medicine and Primary Care, Faculty of Medicine and Health Sciences, Stellenbosch University, Cape Town, South Africa; 2 School of Medicine, Keele University, Keele, United Kingdom

## Asthma and chronic obstructive pulmonary disease in Africa

Chronic respiratory diseases are common in Africa. This group of diseases includes asthma, chronic obstructive pulmonary disease (COPD), post-tuberculosis structural lung damage and pneumoconiosis. Despite being common, they are often relatively neglected and even among non-communicable diseases, diabetes, cardiovascular diseases and hypertension steal the limelight. Given the number of people affected, however, these chronic conditions need to be managed in primary care. Asthma typically affects children, adolescents and younger adults with an atopic background.^1^ In Cape Town, 17% of teenagers in the age group 13–14 years have a history of asthma.^2^ Urbanisation in Africa is one of the key drivers of asthma incidence.^3^ On the other hand, COPD usually affects older adults.^4^ In high-income country settings, COPD is almost always from prolonged exposure to tobacco smoking. While this is also the case in Africa, the picture is complicated by intrauterine growth-related lung problems, exposure to indoor air pollution (burning biomass for heat or cooking), infectious diseases such as human immunodeficiency virus (HIV) and tuberculosis (TB), childhood pneumonia, outdoor air pollution (burning waste or coal) and occupational hazards such as dust exposure in mining.^5^ In Africa, the prevalence of COPD (> 40 years age) is estimated at 13.4% (interquartile range [IQR]: 9.4% to 22.1%).^6^ Managing the condition correctly is dependent on making the correct diagnosis. Primary care providers often treat asthma and COPD as interchangeable diagnoses. In one audit of primary care in the Western Cape as many as 20% of patients had alternating diagnoses.^7^ Fundamentally asthma is a disease of inflammation in the airways that usually has an allergic basis. Airflow restriction is reversible once the inflammation is treated with inhaled corticosteroids (ICS). On the other hand, COPD is a progressive and irreversible disease of the airways and alveoli from exposure to external pollutants. Progression can be prevented, for example by stopping exposure to tobacco smoke or air pollution, and breathing can be assisted by use of inhaled bronchodilators.

## Treatment for asthma

**FIGURE 1:**
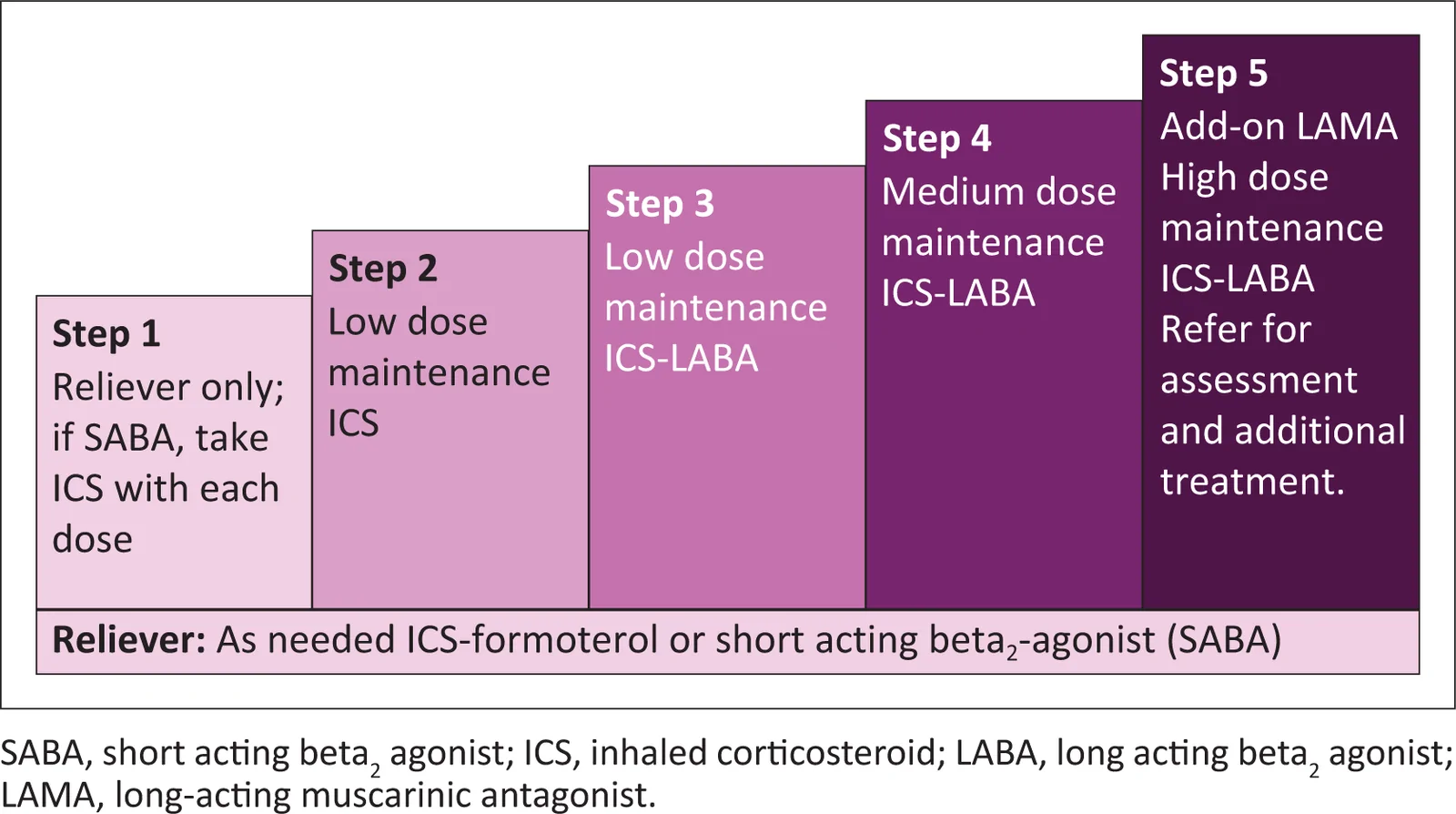
Steps in treatment of asthma.

The Global Initiative for Asthma (GINA) 2025 guidelines recommend a stepwise approach to treatment of asthma with early use of inhaled steroids to treat inflammation and control symptoms.^1^ These guidelines recommend the use of combined ICS-formoterol inhalers which can be used ‘as-needed’ for mild asthma (anti-inflammatory reliever therapy [AIR]) or regularly for moderate or severe asthma (maintenance and reliever regimes [MART]). This ensures patients receive ICS whenever they use their reliever and has the advantage of meaning just one inhaler is needed. However, an alternative approach using separate maintenance and reliever inhalers is also outlined in the GINA guidance, and in the African setting, the steps in Figure 1 are more likely to be feasible. Short acting beta_2_-agonists (SABA) can be used as the reliever but should not be used as monotherapy because they do not address the underlying inflammatory pathology and do not protect against severe exacerbations or even death.^8^

In Africa, however, access to ICS is very limited. Many patients attend pharmacies when they are acutely wheezing and only receive a SABA. Sometimes this is not even an inhaler but syrup or tablets or even oral theophylline, which work slowly and have many other side effects. It is often quicker and more profitable to just give a SABA. Primary care providers may not be well trained in the management of asthma and ICS may not be available in primary care facilities or out of stock. Sometimes patients bypass primary care to attend the district hospital emergency centre, where they may still be treated with a SABA and discharged. It is also possible for patients to receive multiple prescriptions for oral steroids, without ICS, that also have serious side effects if used too often. Pressurised metered dose inhalers (MDIs) should be used with spacers to ensure that the inhaled therapy reaches the lungs, but these are even less likely to be available. Sometimes primary care providers promote making your own spacer from a cool drink bottle. To improve the quality of life of people living with asthma and prevent unnecessary deaths, it is essential that primary care has a reliable supply of ICS and spacer devices; primary care providers must also be able to assess the level of control by asking a few key questions (Table 1), be able to demonstrate and explain how to use an inhaler or spacer, and be able to identify and address common allergens and triggers.^9^ As with all NCDs, patient empowerment is critical. A peak expiratory flow (PEF) meter can be helpful to make a diagnosis (demonstrating reversible airways obstruction) and evaluate the response to acute treatment of an asthma attack. It is not, however, essential to monitor control, which can be determined by questions on symptoms (Table 1).

**TABLE 1:** Assessment of asthma control.

Criteria	Well controlled	Partly controlled	Uncontrolled
Daytime symptoms	≤ twice per week	> twice per week	Three or more features of partly controlled asthma
Night time or early morning symptoms	None	Any	-
Limitation of daily activities	None	Any	-
Need for reliever treatment	< twice per week	> twice per week	-
Exacerbations requiring attention of a healthcare worker	None	> once in last year	-
PEF (% of predicted or best)	≥ 80%	< 80%	-

*Source*: Lalloo U, Ainslie G, Wong M, et al. Guidelines for the management of chronic asthma in adolescents and adults. S Afr Fam Pract. 2007;49(5):19–31. https://doi.org/10.1080/20786204.2007.10873553^10^

PEF, peak expiratory flow.

## Treatment for chronic obstructive pulmonary disease

Chronic obstructive pulmonary disease presents with increasing breathlessness on physical activity, which can be categorised in terms of severity (Table 2 and Table 3).^11,12^ The most important part of treatment is to stop exposure to the external pollutant. Smoking cessation is therefore critical, and primary care providers need skills in brief behaviour change counselling.^13^ Nicotine replacement therapy or pharmacological therapy (e.g. varenicline, bupropion) may be considered in those with high levels of nicotine dependency.^14^ This can be assessed by asking the Fagerstrom questions.^15^

**TABLE 2:** Modified Medical Research Council grades for breathlessness.

Grade	Definition
0	I only get breathless with strenuous exercise
1	I get short of breath when hurrying on the level or walking up a slight hill
2	I walk slower than people of the same age on the level because of breathlessness or I have to stop for breath when walking on my own pace on the level
3	I stop for breath after walking about 100 m or after a few minutes on the level
4	I am too breathless to leave the house or I am breathless when dressing or undressing

*Source:* Adapted from Mahler D, Wells C. Evaluation of clinical methods for rating dyspnea. Chest. 1988;93(3):580–586. https://doi.org/10.1378/chest.93.3.580^11^

**TABLE 3:** Chronic Airways Assessment test.

Lower anchor	0	1	2	3	4	5	Upper anchor
I never cough	-	-	-	-	-	-	I cough all the time
I have no phlegm in my chest at all	-	-	-	-	-	-	My chest is completely full of phlegm
My chest does not fell tight at all	-	-	-	-	-	-	My chest feels very tight
When I walk up a hill or one flight of stairs, I am not breathless	-	-	-	-	-	-	When I walk up a hill or one flight of stairs, I am very breathless
I am not limited doing any activities at home	-	-	-	-	-	-	I am very limited doing activities at home
I am confident leaving my home despite my lung condition	-	-	-	-	-	-	I am not at all confident leaving my home because of my lung condition
I sleep soundly	-	-	-	-	-	-	I don’t sleep soundly because of my lung condition
I have lots of energy	-	-	-	-	-	-	I have no energy at all

*Source*: Adapted from Jones P, Tomaszewski E, Belton L, et al. Validity of the chronic Airways Assessment Test (CAAT) in asthma, asthma+COPD and COPD in NOVELTY. ERJ Open Res. 2025;11(4):1359–2024. https://doi.org/10.1183/23120541.01359-2024^12^

People with COPD are also prone to infective exacerbations. Co-morbidity with cardiovascular disease is also common and driven by tobacco smoking. Pulmonary hypertension from COPD can lead to right sided cardiac failure (cor pulmonale) in severe cases. The focus of treatment is preventing further loss of lung function.^14^ After stopping exposure to the pollutant, it is important to prevent viral and bacterial respiratory infections. Vaccinations can help, such as for influenza, pneumococcus and coronavirus disease 2019 (COVID-19). Maintaining respiratory muscles through physical activity and pulmonary rehabilitation can assist. Avoiding extreme weather events and changes may also help some patients. A few patients may benefit from surgery to remove severely damaged lung tissue, and home oxygen therapy may assist some people with severe COPD. According to the latest Global Initiative for Chronic Obstructive Lung Disease (GOLD) guidelines, initial treatment is with regular use of a long-acting muscarinic antagonist (LAMA) or long acting beta_2_ agonist (LABA) to improve breathlessness.^4^ In people with more severe COPD (mMRC grade ≥ 2 or CAAT score ≥ 10, see Table 2 and Table 3), these can be combined. If someone has two or more moderate exacerbations or one or more hospitalisations in the last 12 months, or if their eosinophil count is ≥ 300 cells/µL, or if they also have features of asthma, then addition of an ICS should be considered.

## Environmental issues with prescribing

**FIGURE 2:**
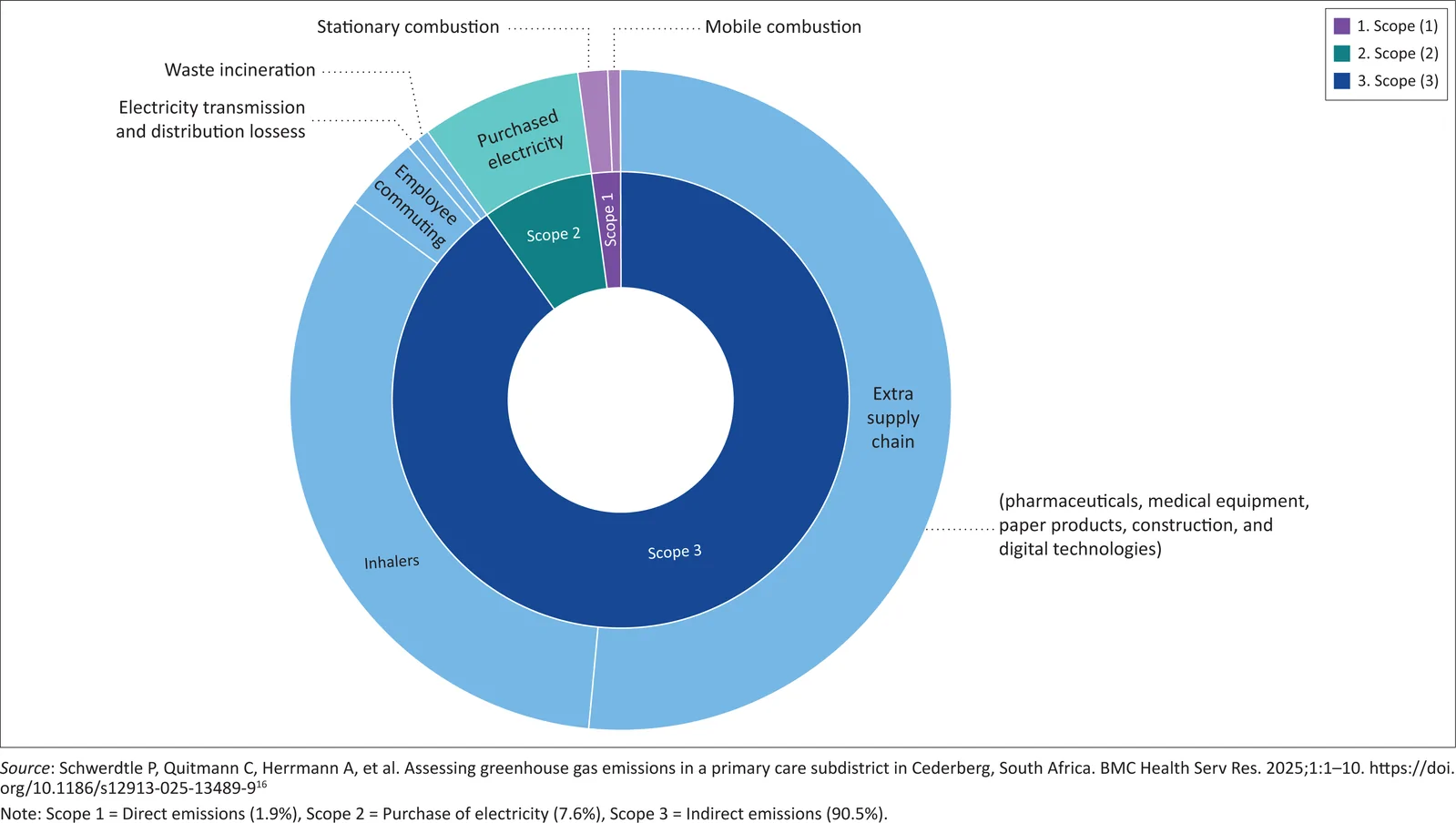
Carbon footprint of primary care facilities in the Cederberg subdistrict, South Africa.

There have been few carbon footprints of health services in Africa. However, a recent study of primary care facilities in the Cederberg subdistrict of South Africa showed MDIs made up 34% of the total footprint (Figure 2).^16^ This is because the hydrofluorocarbon propellants in MDIs are powerful greenhouse gases, estimated to have 12–14 800 times the global warming potential of CO_2_, and hydrofluorocarbons can remain in the atmosphere between 1.4 years and 270 years.^17^ The prescribing of inhalers is therefore associated with a significant contribution to climate change. The increase in temperature, allergens and air pollution from climate change also drives the incidence of chronic respiratory diseases, ironically creating a vicious cycle.

Many commentators on climate change make the point that Africa’s contribution to the problem is relatively negligible. However, future directions are less clear. For example, South Africa’s per capita emissions are already greater than many European countries,^18^ and a recent report found that the carbon intensity of health care in low- and middle-income countries is greater than high income.^19^ In other words, the amount of carbon emitted per dollar spent on health services is higher and emphasises the need to decouple development of health systems from carbon.

## Environmentally responsible prescribing

As we improve access to quality care for chronic respiratory diseases in Africa, there is an opportunity to also reduce the carbon footprint. In asthma, for example, greater use of ICS will lead to better disease control with reduced need for SABA and fewer exacerbations, which will therefore reduce the overall carbon footprint. Well-controlled asthma should have a significantly lower carbon footprint compared to asthma that is not controlled due to reduced use of relievers.^20,21^ Additional approaches that can reduce the carbon footprint from MDIs are to consider prescribing inhalers that have a lower volume of propellant, such as Salamol or Airomir, rather than those with a larger cannister, such as Ventolin, and to consider prescribing fewer puffs of a higher strength inhaler (e.g. 1 puff twice daily of beclomethasone dipropionate 100 mcg instead of 2 puffs twice daily of beclomethasone dipropionate 50 mcg).^22,23^ The use of combination inhalers also reduces the amount of propellent used.^20^ Dry powder inhalers (DPI) are alternative devices that have a carbon footprint that is 18 times lower than MDIs, as they do not contain propellent gases.^24^ Dry powder inhalers rely on a quick, deep inspiration to suck air into the lungs along with the medication and for many people, are easier to use than MDIs. They also do not require a spacer. Provided inhaler technique is adequate, delivery of medication may therefore be better, resulting in better control of asthma and symptomatic relief for COPD. Patients may need reassurance and support in switching from a familiar MDI to a new DPI. In one analysis after 6 months of treatment, the odds of being well controlled in the DPI group were almost twice that of the MDI group.^25^ In addition, the DPI group had fewer reliever inhalers prescribed. Children aged under 7 years are not usually able to use DPIs and an MDI with a spacer will still be needed for this group.

The higher cost of DPIs, however, is a potential barrier to their use. In high-income countries such as United Kingdom (UK), the cost is the same (e.g. Fostair 100/6 the MDI and DPI are both $39.28 according to the British National Formulary), but in a country such as South Africa, DPIs are more expensive and not available in the public sector. The approved drug list in South Africa for the public sector includes only one DPI. Relative costs are for fluticasone (250 mcg)/salmeterol (50 mcg) DPI (60 doses) (R113.85, $6.70) versus fluticasone (125 mcg)/salmeterol (25 mcg) MDI (120 doses) (R74.75, $4.40).^26^ ($1.00 = R17.00 = Great British pound [GBP] £0.75). Improved adherence with DPIs and fewer emergency room visits and hospitalisations may offset these costs within the health system. Nevertheless, the higher cost may preclude procurement in some health systems. In the Western Cape of South Africa, the Department of Health and Wellness has a goal of reducing the carbon footprint of health care by 30% by 2030,^27^ and increasing DPI use in primary care, particularly if ICS–formoterol combination DPIs were available, could help achieve this goal. There is a need to negotiate prices at scale with the pharmaceutical industry and to acknowledge the cost of environmental harm. The pharmaceutical industry is also developing new propellants that have a much lower carbon footprint, but these are still under development. Nevertheless, countries in the European Union have agreed that these changes must be made in the foreseeable future.^28^

## Conclusion

The latest GINA and GOLD guidelines should be adapted for local use in African countries. Treatment of asthma and COPD needs to be supported in essential drug lists, especially the supply of ICS for asthma, and the supply chain should extend to primary care where these conditions need to be managed. Primary care providers need to be trained in the diagnosis, assessment and ongoing care of people with these conditions, including skills and knowledge to empower patients. Improving quality of care can occur alongside reducing carbon emissions through making ICS widely available, using a greater proportion of DPIs and looking to a new generation of MDI propellants.
